# Gradient-free MCMC methods for dynamic causal modelling

**DOI:** 10.1016/j.neuroimage.2015.03.008

**Published:** 2015-05-15

**Authors:** Biswa Sengupta, Karl J. Friston, Will D. Penny

**Affiliations:** Wellcome Trust Centre for Neuroimaging, Institute of Neurology, University College London, 12 Queen Square, London WC1N 3BG, UK

## Abstract

In this technical note we compare the performance of four gradient-free MCMC samplers (random walk Metropolis sampling, slice-sampling, adaptive MCMC sampling and population-based MCMC sampling with tempering) in terms of the number of independent samples they can produce per unit computational time. For the Bayesian inversion of a single-node neural mass model, both adaptive and population-based samplers are more efficient compared with random walk Metropolis sampler or slice-sampling; yet adaptive MCMC sampling is more promising in terms of compute time. Slice-sampling yields the highest number of independent samples from the target density — albeit at almost 1000% increase in computational time, in comparison to the most efficient algorithm (i.e., the adaptive MCMC sampler).

## Highlights

• We compare 4 MCMC sampling methods for inferring parameter distributions in a neural mass model.
• The methods are slice-sampler, random-walk Metropolis–Hastings, adaptive and population MCMC.
• Both adaptive and population MCMC are most promising in terms of computational efficiency.
• We propose combining gradient-based and gradient-free samplers for efficient Bayesian inference.

## Introduction

This technical note reports comparative evaluations of common gradient-free sampling schemes that can be used for Bayesian inference in dynamic causal modelling. It is the first of a series of technical reports that hopes to provide a comprehensive survey of the various sampling schemes available — both gradient-free, and (first and second order) gradient-based schemes. These schemes provide a gold standard against which the performance of fixed form (e.g., variational) approximate Bayesian inference can be compared. Furthermore, with advances in computer science, the computational costs usually associated with sampling schemes may be sufficiently reduced to allow their routine use in applications like dynamic causal modelling.

Dynamical Causal Models (DCMs) are used routinely in neuroimaging as generative models of neurophysiological signals (Friston et al., 2003). Inference on their parameters usually proceeds using a parameterised probability density and maximising the (variational free energy) evidence lower bound (Friston et al., 2007). Typically a Laplace approximation (Tierney and Kadane, 1986) is used for inference in DCMs because it does not require algebraically involved updates, unlike variational Bayes — and is guaranteed to converge as a result of the central limit theorem (Wang and Titterington, 2004). But such deterministic algorithms have their limitations. For example, they underestimate the variability in the posterior density; they get locked in local minima and are unable to approximate multi-modal posteriors (MacKay, 2002). Markov Chain Monte Carlo (MCMC) schemes are stochastic sampling algorithms (Gelfand and Smith, 1990; Geman and Geman, 1984) that eschew these problems. The basic idea behind MCMC is to simulate a Markov chain with the posterior density as its invariant probability density (see Appendix for definitions). After the chain has converged, resulting samples are an approximation of the posterior density.

MCMC methods come in two flavours — gradient-free schemes and gradient-based schemes. Gradient-free methods typically take the form of a Gibbs sampler or some variant of the random walk Metropolis–Hastings algorithm; whilst gradient-based methods use the gradient of the joint log-likelihood function to simulate diffusion (a Langevin algorithm) (Roberts and Tweedie, 1996) or optimise auxiliary variables as in Hamiltonian Monte-Carlo (HMC) algorithm (Neal, 2010). Despite the progress in numerical analysis, gradient-based methods are expensive; however, they avoid the naïve random walk inherent in gradient-free samplers. For both classes of samplers, there exists a natural trade-off — between rapid (Markov) chain mixing versus compute time efficiency. A computationally efficient sampler would reach the invariant probability density quickly (rapid mixing) using least floating-point cycles (computational efficiency). Since the inception of stochastic sampling methods (Gelfand and Smith, 1990; Geman and Geman, 1984) extensive measure theoretic analyses (Meyn and Tweedie, 2009) have been conducted to gauge the mixing properties of Markov chains but these are at worst problem dependent.

In this note, we evaluate the suitability of gradient-free MCMC methods in terms of unit computation required for producing an independent sample from the posterior distribution. For this, we implemented three variants of the Metropolis–Hastings algorithm; along with a slice sampling algorithm. In addition to the standard random walk Metropolis algorithm, we implemented a sampling algorithm that tunes the properties of the proposal distribution; whilst another scheme makes non-local moves across multiple Markov chains at different temperatures — facilitating cross-over between chains. In what follows, the parameters of a (single-node) neural mass model (NMM) were estimated using these inference schemes and their computational efficiency benchmarked. We found that adaptive Monte Carlo methods based on stochastic approximations were the most efficient, followed by MCMC methods based on tempered chains. Discounting computation efficiency, the slice-sampler emerged as a clear winner — if performance was restricted to the number of independent samples they could produce.

## Methods

In this section, we briefly review the generative (dynamic causal) model used to simulate data that was subject to subsequent inference. These models are used to fit observed electrophysiological data and contain between 10 and 100 parameters. We then review the schemes (random walk Metropolis Hastings sampling, slice sampling, adaptive MCMC sampling and population MCMC sampling) that are subject to a comparative evaluation in the Results section.

Custom code was written in Matlab 2014a (The MathWorks Inc., USA) to simulate the Markov chains. For population MCMC sampling Parallel Computing Toolbox (The MathWorks Inc., USA) was used. Unless stated otherwise, out of the 2000 samples that were collected, the initial 600 samples were discarded as burn-in (see Appendix for definitions). In adaptive MCMC, out of the 600 burn-in samples, the initial 300 samples were used for proposal adaptation. All computations were performed on a 2011 Macbook Pro laptop.

### Neural mass models

**Fig. 1:**
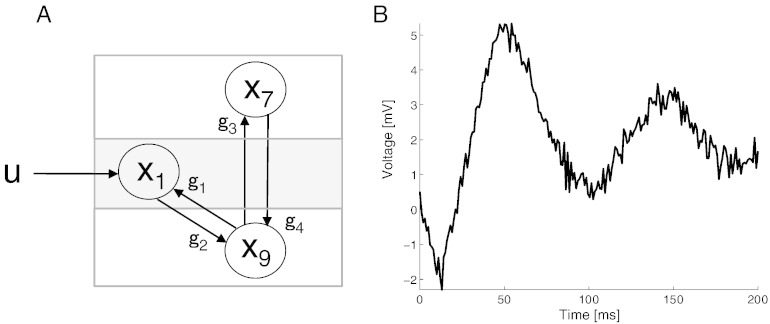
A single node Neural Mass Model (NMM). (A) The forward model consists of 3 neural population — pyramidal (*x*_9_), inhibitory interneuron (*x*_7_) and spiny-stellate cells (*x*_1_) connected by linearised delay links (*g*_1_, *g*_2_, *g*_3_ and *g*_4_) with *u* as a Heaviside input. (B) The pyramidal cell voltage comprises the only observable of the model.

To test the inference schemes under known parameters, we used a single node neural mass model (NMM) based on David et al. (2006) to create synthetic data (Fig. 1). This model comprises ten parameters ({*δ*, *g*, *h*, *τ*, *u*} ⊆ *θ* with *δ* (intrinsic delay), {*g*_1 … 4_} (connection strengths), *h*_*e*/*i*_ (maximum amplitude of post-synaptic potential), *τ*_*e*/*i*_ (rate-constant of the membrane) and *u* (input to the neural population); for detailed description refer to David et al. (2006)) and nine ordinary differential equations (ODEs) that are a first-order approximation of delay-differential equations (DDEs) representing three distinct neural populations; namely, inhibitory interneurons (x_7_), spiny-stellate (x_1_) and pyramidal neurons (x_9_),

(1) $$\begin{array}{l} {x_{1}}^{\cdot }\left(t\right)=x_{4}\left(t\right)\\ {x_{2}}^{\cdot }\left(t\right)=x_{5}\left(t\right)\\ {x_{3}}^{\cdot }\left(t\right)=x_{6}\left(t\right)\\ {x_{4}}^{\cdot }\left(t\right)=\frac{h_{e}\left(g_{1}\left(\frac{1}{e^{-0.56x_{9}\left(t-\delta \right)}+1}-0.5\right)+u\right)}{\tau_{e}}-\frac{x_{1}\left(t\right)}{{\tau_{e}}^{2}}-\frac{2x_{4}\left(t\right)}{\tau_{e}}\\ {x_{5}}^{\cdot }\left(t\right)=\frac{g_{2}h_{e}\left(\frac{1}{e^{-0.56x_{1}\left(t-\delta \right)}+1}-0.5\right)}{\tau_{e}}-\frac{x_{2}\left(t\right)}{{\tau_{e}}^{2}}-\frac{2x_{5}\left(t\right)}{\tau_{e}}\\ {x_{6}}^{\cdot }\left(t\right)=\frac{g_{4}h_{i}\left(\frac{1}{e^{-0.56x_{7}\left(t-\delta \right)}+1}-0.5\right)}{\tau_{i}}-\frac{x_{3}\left(t\right)}{{\tau_{i}}^{2}}-\frac{2x_{6}\left(t\right)}{\tau_{i}}\\ {x_{7}}^{\cdot }\left(t\right)=x_{8}\left(t\right)\\ {x_{8}}^{\cdot }\left(t\right)=\frac{g_{3}h_{e}\left(\frac{1}{e^{-0.56x_{9}\left(t-\delta \right)}+1}-0.5\right)}{\tau_{e}}-\frac{x_{7}\left(t\right)}{{\tau_{e}}^{2}}-\frac{2x_{8}\left(t\right)}{\tau_{e}}\\ {x_{9}}^{\cdot }\left(t\right)=x_{5}\left(t\right)-x_{6}\left(t\right)\text{.} \end{array}$$

These differential equations simulated for *T* time-points provide the predicted response (for some known experimental input) which, assuming additive Gaussian noise with covariance Σ, provide a likelihood model of observed data (*y*) with corresponding log joint density,

(2) $$\begin{array}{l} J=-\frac{1}{2}\ln\left(\left|\Sigma \right|\right)-\frac{T}{2}\ln\left(2\pi \right)-\frac{1}{2}{\left(x_{9}\left(\theta \right)-y\right)}^{T}\Sigma^{-1}\left(x_{9}\left(\theta \right)-y\right)\\ \;+\ln\left(\frac{1}{\Gamma \left(k_{1}\right){k_{2}}^{k_{1}}}\theta^{k_{1}-1}e^{-\frac{\theta }{k_{2}}}\right)\text{.} \end{array}$$

Priors on all parameters (Table 1) conform to a Gamma distribution with shape *k*_1_ and scale *k*_2_, where – by construction – approximately 46–50% of the parameters sampled from this prior result in unstable dynamics, marked by positive real eigenvalues of the Jacobian matrix. This ensured that the inference scheme can recover from dynamical instability. The shape and scale of the Gamma distribution were determined numerically by integrating 200,000 NMMs and performing stability analysis. The shape and scale parameters of the Gamma prior distribution were then chosen, such that 46–50% of the sampled parameters produced unstable dynamics. The fixed-point equations were solved using a Trust-Region Dogleg method (Nocedal and Wright, 2006).

Contrary to David et al. (2006) where experimental input was modelled as a combination of a Gamma density function and a discrete cosine set, we used a simpler Heaviside step function to perturb the spiny-stellate cells. Differential equations were integrated using CVODES (Hindmarsh and Serban, 2002) using implicit backward-differentiation formulas (BDFs). The resulting non-linear equations were solved using Newton's method. Initial simulations established that direct solvers based on dense matrices were computationally more efficient than the three preconditioned Krylov (iterative) solvers (GMRES, Bi-CGStab, and TFQMR) (Golub and Van Loan, 2012). We anticipate that for larger dynamical systems (e.g., a 20-node NMM) iterative solvers may be more efficient. The absolute and relative tolerances of the integrators were both fixed at 10^− 3^.

The source-code will be released as a general purpose ‘Monte-Carlo inference’ toolbox for SPM (http://www.fil.ion.ucl.ac.uk/spm/).

### Algorithm A — slice sampler

Slice-sampling is a type of MCMC based on the fact that sampling a random variable can be attained by sampling uniformly under its probability density function and rejecting those that are outside (Neal, 2003). First of all we initialise our parameters to *θ*_0_ so that the target density *π*(*θ*_0_) > 0. Given this previous sample *θ*_*i*_ we sample a position *n*_*i* + 1_ uniformly on [0, *π*(*θ*_*i*_)]. Conceptually, the next step comprises of drawing a horizontal line across the curve at this position. This hypothetical line is nothing but a ‘slice’ of our target distribution. Consequently, we sample *θ*_*i* + 1_ along the slice so that *π*(*θ*_*i* + 1_) ≥ *n*_*i* + 1_.

Numerically, to operationalise the inequality, a bracket is first constructed as *θ*_*min*_ ≤ *θ*_*i* + 1_ ≤ *θ*_*max*_ and tested to see whether each end point lies within the slice. If it does, the endpoint is extended in that direction until it is outside the slice. This process is called “stepping out”. A candidate value $\tilde{\theta }$ is then selected uniformly from the region — and is accepted as the next sample if it lies within the slice i.e., $\theta_{i+1}=\tilde{\theta }$. If not, the slice shrinks, such that $\tilde{\theta }$ forms one end of the slice containing *θ*_*i*_. The process is repeated until a sample is accepted. For multivariate distributions, we introduce an auxiliary position (*n*) for each dimension.

Unlike a Gibbs sampler, sampling using slices of the distribution does not require specification of the full conditionals. Similarly in contrast to a Metropolis–Hastings sampling algorithm, a slice-sampler does not require specification of a proposal distribution.

### Algorithm B — random walk metropolis sampler

The random walk Metropolis (RWM) is the most common MCMC algorithm for Bayesian inference. Given a current value *θ*_*i*_ of a *d*-dimensional Markov chain, the next value is chosen according to a proposal distribution $\tilde{\theta }\sim \pi \left(\tilde{\theta }\left|\theta_{i}\right)\right)$. We choose this to be a multi-variate Gaussian. The sample is then accepted with probability,

(3) $$\alpha =1\wedge \frac{\pi \left(y\left|\tilde{\theta }\right)\right)\pi \left(\tilde{\theta }\right)\times \pi \left(\theta \left|\tilde{\theta }\right)\right)}{\pi \left(y\left|\theta \right)\right)\pi \left(\theta \right)\times \pi \left(\tilde{\theta }\left|\theta \right)\right)}\text{.}$$

∧ denotes minimum between the left and the right arguments. If *z* ≤ *α* where *z* ~ *U*(0, 1) we set $\theta_{i+1}=\tilde{\theta }$. Otherwise, we set *θ*_*i* + 1_ = *θ*_*i*_. The above formula embodies the notion that any proposal that takes the chain closer to a local mode is always accepted, whilst any other proposal is accepted with the probability equal to the relative densities of the posterior at the proposed and the current values.

### Algorithm C — adaptive MCMC sampler

The random walk Metropolis (RWM) scheme generally has a slow convergence to the target density because of the inherent random walks (Chumbley et al., 2007). Using the history of samples that are generated from a Markov chain, the adaptive MCMC algorithm (Andrieu and Thoms, 2008; Haario et al., 2001) adapts the expectation and covariance matrix of the proposal distribution using stochastic approximations (Kushner and Yin, 2003). Stochastic approximation is an iterative algorithm that finds extrema (roots) of cost–functions using noisy samples. In adaptive MCMC this cost–function is based on the empirical mean (*μ*) and covariance (Σ) of the target density as well as the pre-determined acceptance rate (to update the scalar scale parameter *λ*).

Specifically, a Robbins–Monro algorithm is used (Robbins and Monro, 1951); wherein given current parameters (*θ*_0_), mean (*μ*_0_) and covariance (*Σ*_0_) of the proposal distribution we first sample $\theta_{i+1}\sim N\left(\mu_{i},\lambda_{i}\Sigma_{i}\right)$ where *λ*_0_ is initialised to 1. Similarly, *Σ*_0_ was initialised to an identity matrix. Secondly, using the Metropolis–Hastings criteria (Eq. (3)) we set *μ*_*i* + 1_ = *θ*_*i* + 1_. If not, we reject the sample and set *μ*_*i* + 1_ = *μ*_*i*_. The current and target acceptance probabilities are *α*_*i* → *i* + 1_ and *α*_*target*_, respectively. It may be easy to understand such a scheme as a stochastic realisation of a deterministic prediction-error learning rule, guided by the Metropolis–Hastings acceptance ratio.

For the subsequent iteration, we adapt the mean (*μ*), the covariance (*Σ*) and the global scale of the covariance matrix (*λ*) with an iteration dependent step-size (*γ*) as follows,

(4) $$\gamma_{i+1}=\frac{1}{i+1}$$

(5) $$\begin{array}{c} \log\left(\lambda_{i+1}\right)=\log\left(\lambda_{i}\right)+\gamma_{i+1}\left[\alpha_{i\to i+1}-\alpha_{\mathrm{target}}\right]\\ \mu_{i+1}=\mu_{i}+\gamma_{i+1}\left[\theta_{i+1}-\mu_{i}\right]\\ \Sigma_{i+1}=\Sigma_{i}+\gamma_{i+1}\left[\left(\theta_{i+1}-\mu_{i}\right){\left(\theta_{i+1}-\mu_{i}\right)}^{T}-\Sigma_{i}\right]\text{.} \end{array}$$

### Algorithm D — population MCMC sampler

In the two preceding MCMC schemes, one simulates a single Markov chain, where the posterior sample density is said to have converged if multiple starting points yield identical invariant distributions. Slow chain mixing results from non-convexity of the posterior density. In order to promote chain mixing, one can run multiple chains with varying temperatures and implement non-local proposal swaps between chains (Geyer, 1992a). These exchanges also make the algorithm a candidate for sampling from multimodal densities (Frantz et al., 1990). Such an algorithm is known as the population MCMC sampler (Geyer, 1992a). It has been re-invented under various guises (Replica Exchange (Swendsen and Wang, 1986), Metropolis-Coupled MCMC (Geyer, 1992a), population MCMC (Laskey and Myers, 2003), Parallel Tempering (Earl and Deem, 2005), among others) but the standard approach is to initiate multiple Markov chains (indexed by i) totalling *N* such that the inverse temperature (*β*_i_) of each chain is distributed according to

(6) $$\beta_{i}=1-{\left(\frac{i}{N}\right)}^{p}$$

where *p* = 5 and *N* = 4 chains, with the posterior density specified as,

(7) $$\pi \left(\left(\theta \right|y\right)\propto \pi {\left(\left(y\right|\theta \right)}^{\beta }\pi \left(\theta \right)\text{.}$$

With a *β* of 1, the chain represents the joint log-likelihood, whilst a *β* of 0 represents a Markov chain that samples from the prior — the lower the inverse-temperature, the smoother the posterior density. One can visualize the multi-modal target distribution melting with an increase in temperature. At each temperature, the resulting distribution is explored using a single Markov chain whilst a product distribution is considered when moving between individual chains. This enables the sampler to take into account all of the chains, at different temperature levels.

Operationally, each chain uses a local (to that chain) Metropolis–Hastings acceptance criterion to either accept or reject the sample i.e., *z* < (1 ∧ exp(H_old_ − H_new_)) where *z* ~ *U*(0, 1) and H is the unnormalised joint log-likelihood. After the *k*th sample is collected from the local chain, an additional Metropolis–Hastings acceptance criteria is imposed — whereby a pair of chains (*t*_*i*_ and *t*_*j*_) is selected randomly and their samples are swapped with the following acceptance ratio,

(8) $$1\wedge \frac{L{\left(y\left|\theta_{j}\right)\right)}^{t_{i}}\times L{\left(y\left|\theta_{i}\right)\right)}^{t_{j}}}{L{\left(y\left|\theta_{i}\right)\right)}^{t_{i}}\times L{\left(y\left|\theta_{j}\right)\right)}^{t_{j}}}$$

L is the log-likelihood of the predicted response. It is customary to use a uniform tempering schedule; i.e., $\beta_{i}=\frac{i}{N}$ as discussed in Jasra et al.(2007). For linear regression models Calderhead and Girolami (2009) showed that a power law distribution for the tempering schedule is most optimal. They showed that using uniformly spaced temperature schedule on the other-hand produced worse results, even if the number of Markov chains is increased ten-fold. Therefore, in this paper, we evaluate the sampling efficiency as that obtained by both uniform and power-law temperature schedules.

Population MCMC is reminiscent of an embarrassingly parallel problem; wherein communication between multi-threaded processes is minimised by performing proposal exchange only after *k* (fixed at 10) iterations.

This concludes our brief description of the gradient-free sampling schemes considered in this paper.

## Results

We used a single node neural mass model (NMM, Fig. 1) to characterise the computational performance of four different MCMC sampling algorithms, for parameter inference. Inference was performed under the assumption that the (neuronal) system is partially observable i.e., only the pyramidal cell voltage (*x*_9_ in Eq. (1)) was available.

The efficiency of a MCMC sampler is defined as the ratio of the computation time and the number of effective samples produced in that time. The effective sample size (ESS) for each parameter is calculated using $ESS=R{\left\{1+2\sum_{q}\gamma \left(q\right)\right\}}^{-1}$, where *R* is the number of posterior samples post-burn-in and $\sum_{q}\gamma \left(q\right)$ is the sum of *Q* monotonic auto-correlations. This auto-correlation is estimated using the initial monotone sequence estimator (Theorem 3.1 in Geyer (1992b)). The minimum ESS reports the number of samples that is effectively uncorrelated over all parameters. Similarly, the time normalised (wall-time/minimum ESS) ESS tells us how much time we spend sampling a single uncorrelated sample, providing us with a measure of the worst-case behaviour (Cormen et al., 2001) of the sampling algorithm. In short, an efficient sampler would produce a large ESS at the shortest possible time.

**Fig. 2:**
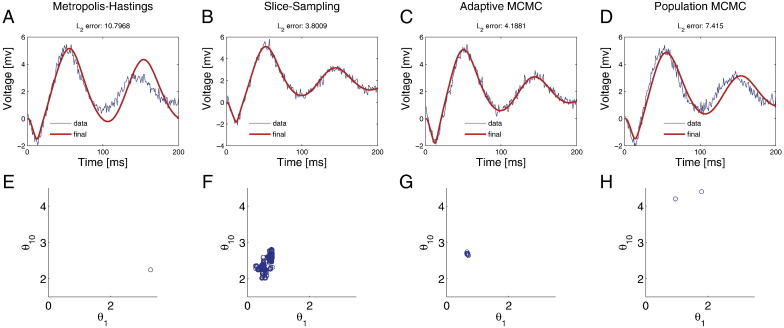
Efficiency of the MCMC methods. (A) Predicted voltage using the posterior mean computed from 1400 samples based on random walk Metropolis–Hastings algorithm. (B) Same as A but with the slice-sampling algorithm. (C) Same as A but with adaptive Metropolis algorithm based on stochastic approximations. (D) Same as A but with population Metropolis algorithm based on proposal exchange. (E) Schematic displaying (effective) samples drawn from the posterior density using the MH algorithm. Parameters 1 and 10 are plotted. (F) Same as E but using the slice-sampling algorithm. (G) Same as E but using the adaptive Metropolis algorithm. (H) Same as E but using the population Metropolis algorithm.

We used a normal symmetric random walk Metropolis (RWM) scheme — as used in previous work on sampling schemes for DCM by Chumbley et al. (2007). The *l*_2_ error-norm was among the highest over all schemes considered (10.8) (Fig. 2A, E). The RWM algorithm resulted in the lowest ESS (Table 2). This is because chain mixing is confounded by the inherent random walk displayed by this class of algorithm. The slice-sampler on the other hand had the highest ESS, albeit at increased computational cost (Fig. 2B, F). Also, it had the lowest *l*_2_ error (Table 2).

In terms of computational time, adaptive Metropolis (Fig. 2C, G) with stochastic approximations of the mean, covariance and the scale of the proposal distribution emerged as the clear winner (Table 2). It had lower ESS in comparison to slice-sampling but took 90% less time to produce a single independent sample. The increased ESS reflects the fact that – unlike the RWM – the proposal distribution has been adapted to guarantee a prescribed acceptance rate of 23%.

So far we have only considered a single Markov chain. Multiple chains running at a variety of temperatures can be used to not only facilitate rapid chain mixing but also to sample from multi-modal posterior densities (Fig. 2D, H). At about 150% increase in compute time (with respect to adaptive MCMC), the population Metropolis method (with 4 chains running at 4 different temperatures with proposal swaps every 10 iterations) has the highest ESS after slice-sampling, but with a 6-fold decrease in compute time per independent sample. This is dependent upon the temperature spacing of the parallel chains; where uniform spacing of inverse temperature performs poorly (Table 2).

Adaptive Metropolis and population based Markov chains appear to be equally efficient; although the latter requires expert intervention in choosing the number of chains, the form of inverse temperature ladder and the selection of proposal exchange partners. Adaptive Metropolis on the other hand did not have any parameters that require tuning. In summary, for inversion of these sorts of DCMs, our (gradient-free) sampler of choice is the single chain adaptive MCMC algorithm.

## Discussion

In this note, we compared four gradient-free MCMC methods — random-walk Metropolis sampler, slice-sampler, adaptive MCMC sampler and population-based MCMC sampler in terms of their effective sample size (ESS). Both adaptive and population MCMC take between 0.4 and 0.6 min (on a 2011 Macbook Pro laptop) to generate a single uncorrelated sample. Adaptive MCMC does this by matching the proposal density to the required target density, whilst proposal-exchanges enable neighbouring chains to mix more quickly in population MCMC sampling. This is particularly useful for DCMs, where such population of Markov chains enable the inference algorithm to ameliorate issues like local minima and multi-modal posterior densities that are characteristic of many variational algorithms. The population MCMC that we have used is similar to a genetic algorithm (GA), where the samples from different chains interact to mimic natural selection. The key difference is that GAs find a single optimum point, whilst population MCMC furnishes a probability density. The temperature ladder can be seen as a cross-over process where fitter samples move to a lower temperature.

Poor-mixing results when the Markov chain is confined to isolated modes or mix poorly along samples with strong correlations. Indeed using multiple chains allows for a mode hopping characteristic for the underlying Markov chain. This is especially useful when sampling from multimodal posteriors. This is because non-local moves are made that result in crossing the barrier imposed by a local potential well. In addition to the population estimator that we have evaluated, a multitude of mode-hopping MCMC samplers exist for tortuous posterior densities. This ranges from Jump-walking (J-Walking) estimator (Frantz et al., 1990) with a potential problem of not satisfying detailed balance to Smart-darting (S-darting) where detailed balance of population walkers is maintained (Andricioaei et al., 2001).

In neuroimaging, the practitioner is not only interested in estimating the distribution of parameters that explain the EEG, MEG or fMRI signal but also build a variety of models to test competing hypothesis about the same observed data. Population MCMC – in the form of power posteriors – not only helps in parameter inference but also facilitates estimation of the partition function or model evidence. Via thermodynamic integration (Friel and Pettitt, 2008; Lartillot and Philippe, 2006) the model evidence can be obtained by running multiple Markov chains in parallel and numerically integrating samples over a variety of temperatures (using quadratures) to compute the model evidence (Eqn. 15 in Calderhead and Girolami (2009)). This becomes important when one has to choose between models using model comparison (Claeskens and Lid Hjort, 2008). Standard MCMC technology that relies on unnormalised probability densities infers the model evidence from samples generated by independent chains. Unlike thermodynamic integration, such estimates of the model evidence are highly variable — even in the high sample-size limit (Calderhead and Girolami, 2009; Jasra et al., 2007), rendering Bayesian model comparison useless. In population MCMC, since multiple chains take about the same amount of time as single chains due to the inherent parallelism of the scheme, one can evaluate model evidence without additional cost.

Unlike Lartillot and Philippe (2006) who suggest a uniform schedule for the temperature ladder, we verified that using a temperature ladder with power-law characteristics was beneficial (in terms of ESS) as pointed out by Calderhead and Girolami (2009). Such a schedule is not only beneficial from the point of the ESS but also in reducing the variance of the model evidence (Calderhead and Girolami, 2009). This is because geometric schedules – that model power-law densities – are the extremal solutions of the Monte Carlo variance (Gelman and Meng, 1998).

Our inference algorithm based on adaptive MCMC sampler adapted the proposal distribution only during the burn-in iterations. This was done to avoid using past information infinitely often, preserving the Markov property of the transition kernel. An alternate methodology adopted by Gelfand and Sahu (1994) is to run several chains in parallel and use sampling-importance-resampling (SIR) (Rubin, 1998) to form kernels that have higher ESS whilst suppressing those chains that do not, using the approximation to the marginal distribution of the chain as a proposal distribution. It is vital to keep in mind that continued adaptation can disturb the invariant distribution of the chain. Although computationally inefficient, adaptation using delayed rejection (Tierney and Mira, 1999) or regeneration (Gilks et al., 1998) can be helpful.

An important issue – when using MCMC for Bayesian inference – is determining when the chain has converged. This criterion is crucial and therefore forms a large part of ongoing research that ascertains rapid convergence. Running an ergodic sampler for an infinite amount of time will result in convergence on the ground truth, per definition — tougher convergence criteria can necessitate longer runtimes. Measure-theoretic analysis of most MCMC samplers gives an estimate of the number of samples required to ensure convergence, according to a total variation distance (with a specified tolerance bound) to the true posterior density. For empirical problems this is seldom possible. A simpler but computationally wasteful strategy involves running multiple – yet independent – chains and ensuring that the posterior density obtained by each chain is identical in terms of its lower moments. A more cogent diagnostics to estimate convergence of the Markov chain uses the normal theory approximations of Gelman and Rubin (1992). This introduces a shrink factor that tends to 1 (depending on between chain and within chain convergence) as the Markov chain converges. For a discussion of convergence estimators, see Table 1 in Cowles and Carlin (1996).

There is no one sampler that is suitable for all inference problems; MCMC samplers that are based on geometric formulation of gradients, adaptation and tempering/annealing have over the years reduced concerns about local minima and sampling of multi-modal posteriors typically faced by deterministic algorithms. An important future development would be in terms of combining gradient-free MCMC estimators with their gradient-based counterparts. For NMMs, we notice that the gradient based manifold Langevin samplers are computationally efficient (Sengupta et al., under review); yet the ESS is not as high as the Hamiltonian Monte Carlo (HMC) sampler. Thus, a gradient-based algorithm can be used as a starting algorithm followed with a population gradient-free MCMC sampler. The proposal distribution of each chain could be individually adapted, forming an adequate trade-off between computational time and the number of independent samples.

## Figures and Tables

**Table 1:** Model parameters used for dynamic causal modelling. Parameters describing the prior Gamma distribution. Also shown are the parameters for generating the ground truth (Fig. 1).

Parameter	Shape (*k*_1_)	Scale (*k*_2_)	True parameters
*g*_1_	18.16	0.03	0.42
*g*_2_	29.9	0.02	0.76
*g*_3_	29.14	0.005	0.15
*g*_4_	30.77	0.007	0.16
*δ*	22.87	0.51	12.13
*τ*_*i*_	34.67	0.23	7.77
*h*_*i*_	20.44	0.96	27.88
*τ*_*e*_	33.02	0.16	5.77
*h*_*e*_	24.17	0.07	1.63
*u*	23.62	0.13	3.94

**Table 2:** Effective sample size (ESS) obtained from various samplers. Wall-time and average ESS for 10 parameters. Worst-case time normalised ESS is computed using the minimum ESS for each method.

Sampler	Time (minutes)	Mean ESS (samples)	Time/min ESS (minutes/smpl)	*l*_2_ error
Slice sampler	11.8	7.23	3.68	3.8
Metropolis–Hastings	1.22	1	1.22	10.8
Adaptive Metropolis	1.06	4.07	0.38	4.2
Population Metropolis (power)	2.67	4.47	0.59	7.4
Population Metropolis (uniform)	2.61	1	2.61	8.6

## Appendix A Basic MCMC terminology

Invariant distribution: A probability distribution *π* is called invariant if and only if πT=T i.e., *π* is a left eigenvector for T with eigenvalue 1. T is the transition kernel (a conditional probability).
Criteria for an invariant distribution: For the distribution of *θ*_*t*_ to converge to its invariant or stationary distribution, the Markov chain has to be (a) irreducible — starting from any point the chain can reach any non-empty set with positive probability (also known as probabilistic connectedness condition), (b) aperiodic — returns to a state at irregular times; this stops the chain from oscillating and (c) positive recurrent — if the initial *θ*_0_ is sampled from *π*(⋅) then all subsequent iterates will also be distributed according to *π*(⋅).
Ergodicity of the Markov chain: A state is ergodic if it is aperiodic and positive recurrent, which means that the state has a period of 1 and has finite average recurrence time. If all states of a (irreducible) Markov chain are ergodic, then the chain is said to be ergodic. Consequently, a Markov chain will have a unique invariant probability density (in our case the approximate posterior density) if and only if the states are positive recurrent.
Geometric ergodicity: The distribution of *θ* is geometrically ergodic in total variation norm if it is (a) ergodic and (b) there exists a *κ* in [0, 1) and a function V>1 s.t. $\sum_{j}\left|T_{ij}\left(t\right)-\pi \left(j\right)\right|\leq V\left(i\right)\kappa^{t}$. The smallest *κ* for which the function V exists is called the rate of convergence.
Uniform ergodicity: An ergodic Markov chain is uniformly ergodic if there exists a finite constant V and a *κ* in [0,1) s.t. $\sum_{j}\left|T_{ij}\left(t\right)-\pi \left(j\right)\right|\leq V\kappa^{t}\text{.}$
Convergence of RWM: A symmetric random walk Metropolis Hastings (RWM) algorithm cannot be uniformly ergodic when the state space is not bounded (see Theorem 3.1 and 3.2 in Mengersen and Tweedie (1996)), although it can be geometrically ergodic. Geometric ergodicity is equivalent to the acceptance probability being uniformly bounded away from zero.
Burn-in: Burn-in refers to the practice of discarding initial iterations of a Markov chain to specify initial distributions of the form $\pi T^{l}$. *l* is the number of burn-in iterations. Note that the strong law of large numbers and the central limit theorem holds regardless of the starting distribution.
